# Shape analysis of sand particles based on Fourier descriptors

**DOI:** 10.1007/s11356-023-26388-5

**Published:** 2023-03-22

**Authors:** Tianxi Yan, Yahong Liu, Dong Wei, Xiaofan Sun, Qing Liu

**Affiliations:** 1 School of Water Resources and Environment, China University of Geosciences, Beijing, 100083 China; 2 School of Earth Sciences and Resources, China University of Geosciences, Beijing, 100083 China; 3 Institute of Hydrogeology and Engineering Geology of Hubei Province, Yichang, 443100 China; 4 Seventh Geological Brigade of Hubei Geological Bureau, Yichang, 443100 China; 5 Yichang Geological Environment Monitoring Station, Yichang, 443100 China

**Keywords:** Sand particle, Shape analysis, Fourier descriptors, Reconstruction, Correlation coefficients

## Abstract

Particle shape greatly influences the mechanical behavior of geotechnical materials. For a specific material, for example, a sand particle, it remains an open question how to provide a comprehensive quantitative information about the particle shape. While Fourier descriptors, as a sequence of good shape descriptors, are well addressed in the literature, approaches mainly aim at pattern recognition in the field of computer vision. In this paper, Fourier descriptors are used to analyze the particle shape of geotechnical material. A total of 600 sand particles were collected from the Hutuo River, the main water resource of Shijiazhuang County, Hebei Province. Several shape descriptors, elongation, circularity, convexity, and roughness, are identified and further analyzed statistically. The Andrews plot of Fourier descriptors can be used to discriminate the sand samples. And it is convenient to use Fourier descriptors to reconstruct sand particles. A functional relationship between Fourier descriptors *D*_*k*_ and *k*, the frequency of the *k*th components is proved to exist. Moreover, the relationship between shape descriptors and Fourier descriptors is investigated in a correlation analysis. The elongation has a moderate correlation with Fourier descriptors of *D*_*1*_.

## Introduction

The discrete element method (DEM) provides great convenience to model granular materials (such as sand, soil, rockfill, and debris flow) in geotechnical field (Bu et al. 2022; Xu et al. 2021; Xu and Dong 2021). However, the conventional DEM models use disks to replace the irregular particles (Gao et al. 2021). In fact, it has been proved by many studies that the morphological signatures of sand particles have a considerable impact on the mechanical behavior (such as friction, strength, dilation, compressibility, and crushability) (Guo and Su 2007; Tsomokos and Georgiannou 2010; Altuhafi and COOP 2011; Yang and Luo 2015). For example, the interlocking phenomena observed in sand have been generally attributed to the irregularity and angularity and considered to be closely related to the strength and dilatancy of sand particles (Mair et al. 2002; Suh et al. 2017). Thus, accurate shape analysis is pivotal to obtain deep understanding towards the complicated mechanical behavior of sand particles.

In order to quantify the particle shape, a set of shape descriptors (such as elongation, circularity, roundness, roughness, and convexity index) were adopted (Suhr et al. 2020; Suhr and Six 2020; Das 2007). Each of these shape descriptors only quantifies a single aspect of the geometry features of the particle and has limitation to provide comprehensive quantitative information about the particle morphology. The Fourier descriptors have been proved to be simple and efficient in describing and recognizing the object shape (Wang et al. 2021; Sokic and Konjicija 2016). Meloy applied the fast Fourier transform (FFT) to analyze the shape of particle silhouettes and postulated that the particle signatures were dependent on the Fourier descriptors and not on the phase angles (Meloy 1977). Mollon illustrated the qualitative and quantitative relationship between the Fourier descriptors and the particle morphological features and also pointed out that the variation in the phase angles resulted in changes of particle shape (Mollon and Zhao 2012).

The specific objectives of this study were to examine the effects of using Fourier descriptors to analyze and reconstruct sand particle boundary and to investigate the relationship between regular shape descriptors (such as elongation, circularity, convexity, and roughness) and Fourier descriptors. The study was conducted in the form of a case study of 600 grains of sand particle collected from the Hutuo River, the main water resource of Shijiazhuang County, Hebei Province. Firstly, shape descriptors of sand samples, including elongation, circularity, convexity, and roughness, were calculated, and statistical features were analyzed. Secondly, Fourier descriptors were obtained, and the Andrews plots of Fourier descriptors were used to discriminate the sand samples. And then, a method to reconstruct sand particles using Fourier descriptors was proposed. Lastly, a set of Fourier-descriptor-controlled experiments was conducted to research the relationship between shape descriptors and Fourier descriptors qualitatively. Furthermore, several 3D-scatter plots and Pearson’s coefficient were used to describe the correlation between shape descriptors and Fourier descriptors quantitively.

All the algorithms including image processing and data analysis were written specifically for this study and were executed on Matlab 2022-a.

## Descriptors of particle shape

### Shape descriptors

**Fig. 1:**
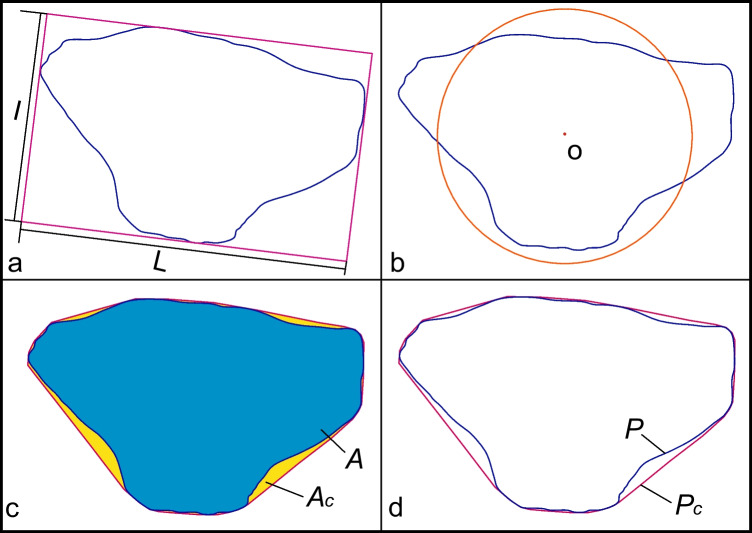
Shape descriptors of an illustrate particle **a** elongation = 0.6448, **b** circularity = 0.7959, **c** convexity = 0.9485, **d** roughness = 0.9935

Although a variety of shape descriptors have been proposed in the literature, elongation, circularity, convexity, and roughness are most often adopted in related research. The elongation (Fig. 1a) is defined below in Eq. (1), denoting by I and L, the shortest and longest axes of the particle’s minimum bounding box (Suhr and Six 2020).

1 $$\mathrm{Elongation}=I/L$$

The particle circularity (Fig. 1b) may be defined as the ratio of the circumference of a circle of the same area as the particle to the actual circumference of the particle (Das 2007). The standard equation to calculate circularity is

2 $$\mathrm{Circularity}= 4\pi A/{P}^{2}$$

where *A* is the area of the particle, and *P* is the perimeter of the particle. The convexity (Fig. 1c) follows the definition:

3 $$\mathrm{Convexity}=A/{A}_{c}$$

where *A* is the area of the particle, and *Ac* is the area of the convex hull (Yang et al. 2019). The roughness (Fig. 1d) of a particle is defined as

4 $$\mathrm{Roughness}=P/{P}_{c}$$

which is the ratio of the particle perimeter to the convex perimeter (Janoo 1998).

### Fourier descriptors

**Fig. 2:**
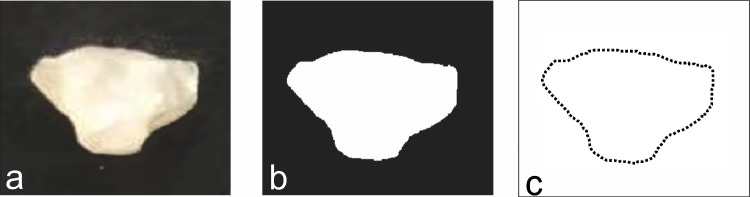
Image processing **a** original particle image, **b** binarization, **c** edge detection

Fourier descriptors are the Fourier transform coefficients, actually the amplitudes of spectrum computation of particle silhouettes. Image processing is necessary to obtain the coordinates of the points on the particle silhouettes before Fourier descriptors are calculated, which consists of image denoising, image binarizing, edge detection, and lastly returning *x*–*y* coordinates of contour points (see Fig. 2).

The *x*–*y* coordinates of N contour points can be treated as a set of complex numbers so that

5 $$s\left(n\right)=x\left(n\right)+iy\left(n\right), n= 0, 1, 2, \dots , N-1$$

That is, the *x*-axis is treated as the real axis and the *y*-axis as the imaginary axis of a sequence of complex numbers. Although the interpretation of the sequence is restated, the nature of the boundary itself is not changed. Of course, this representation has one great advantage: it reduces a 2-D to a 1-D description problem (Gonzalez 2009). The discrete Fourier transform (DFT) and the inverse Fourier transform (IFFT) are

6 $$\left\{\begin{array}{c}z\left(k\right)= \sum_{n=0}^{N-1}\left(x\left(n\right)+iy\left(n\right)\right){e}^{-i\frac{2\pi }{N}kn}\\ s\left(n\right)= \frac{1}{N}\sum_{k=0}^{N-1}z\left(k\right){e}^{i\frac{2\pi }{N}kn}\end{array}\right.$$

where *k* = 0, 1, 2, …, *N* − 1; *n* = 0, 1, 2, …, *N* − 1. The Fourier descriptors are defined as

7 $$d\left(k\right)=\left|\left|z\left(k\right)\right|\right|, k= 0, 1, 2, \dots , N-1$$

in which ||**·**|| means calculating the absolute value of a complex number. The Fourier descriptors should be as insensitive as possible to translation, rotation, and scale changes. Hence, the normalized Fourier descriptors *D*_*k*_ are proposed in order to remove the influence of rotation, translation, and scale changes of the particle silhouettes on Fourier descriptors. From basic mathematical analysis, rotation can be considered by an angle *φ*, translation by a displacement {Δx0, Δy0}, and scale changes by *r* times, and the new Fourier coefficients should be

8 $$\left\{\begin{array}{c}{z}^{^{\prime}}\left(0\right)=r{e}^{i\varphi }z\left(0\right)+F\left({x}_{0}+i{y}_{0}\right), k=0 \\ {z}^{^{\prime}}\left(k\right)=r{e}^{i\varphi }{e}^{i\frac{2\pi }{N}ka}z\left(k\right), k=1, 2,\cdots ,N-1\end{array}\right.$$

and

9 $$\frac{\left|\left|{z}^{^{\prime}}\left(k\right)\right|\right|}{\left|\left|{z}^{^{\prime}}\left(1\right)\right|\right|}= \frac{r\left|\left|{e}^{i\varphi }{e}^{i\frac{2\pi }{N}ka}z\left(k\right)\right|\right|}{r\left|\left|{e}^{i\varphi }{e}^{i\frac{2\pi }{N}ka}z\left(1\right)\right|\right|} \equiv \frac{\left|\left|z\left(k\right)\right|\right|}{\left|\left|z\left(1\right)\right|\right|}$$

Thus, rotation and scale changes simply affect all coefficients equally by a multiplicative constant term $r{e}^{i\varphi }$. Note that the translation only affects *D*_*0*_ and has no effect on the other descriptors *D*_*k*_ for *k* > 0, so that the first descriptor *D*_*0*_ can be set to zero. Finally, the normalized Fourier descriptors are defined as

10 $$D\left(k\right)=\frac{\left|\left|z\left(k\right)\right|\right|}{\left|\left|z\left(k\right)\right|\right|} , k= 1, 2, \dots , N-1$$

**Fig. 3:**
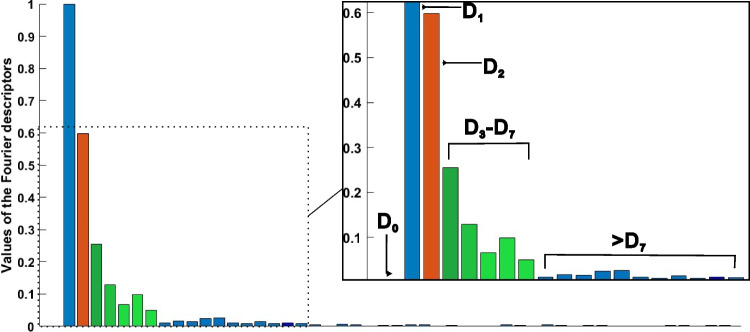
Illustrative normalized Fourier descriptors

An illustration of Fourier descriptors of the particle in Fig. 2 is shown in Fig. 3. According to the Fourier transform, low-frequency components determine the overall shape of particles, and high-frequency components account for fine detail (Gonzalez 2009). As Fig. 3 shows, the descriptors *D*_*k*_ when *k* > 18 are almost equal to zero. Moreover, the particle boundary can be reconstructed using Fourier descriptors by the IFFT in Eq. (8).

## Materials

**Fig. 4:**
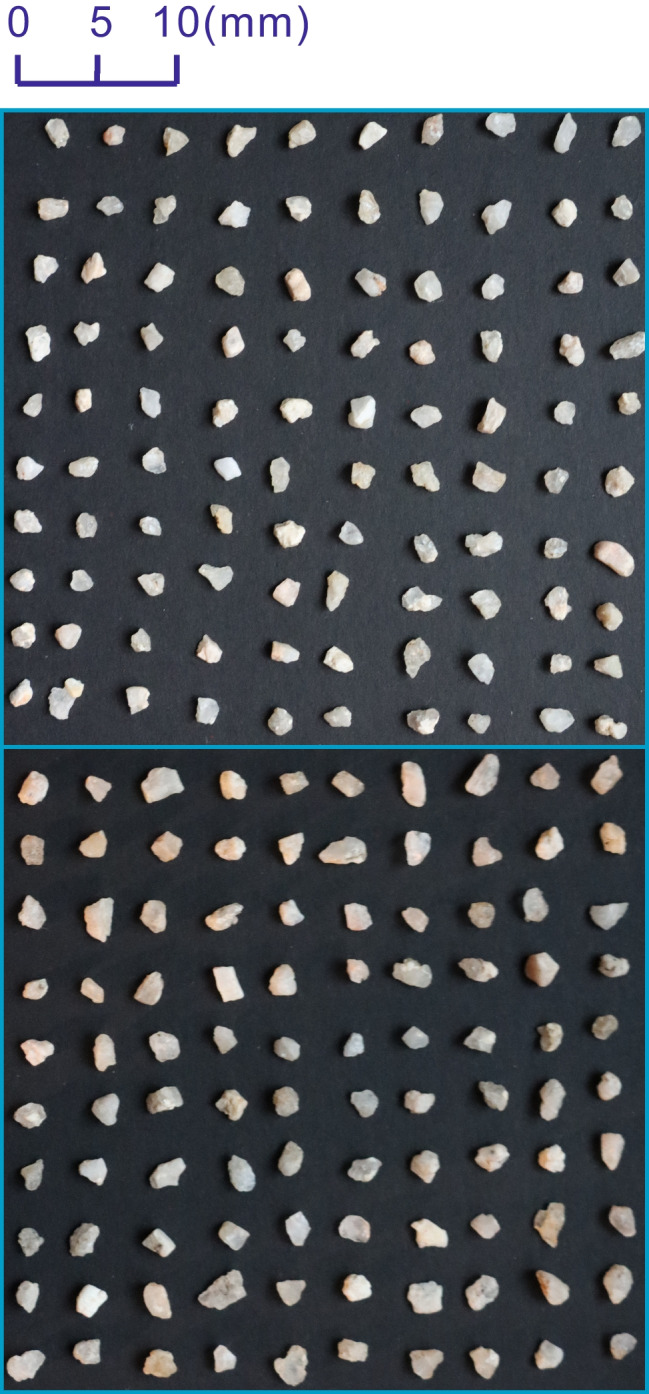
Sand sample of RSI

**Fig. 5:**
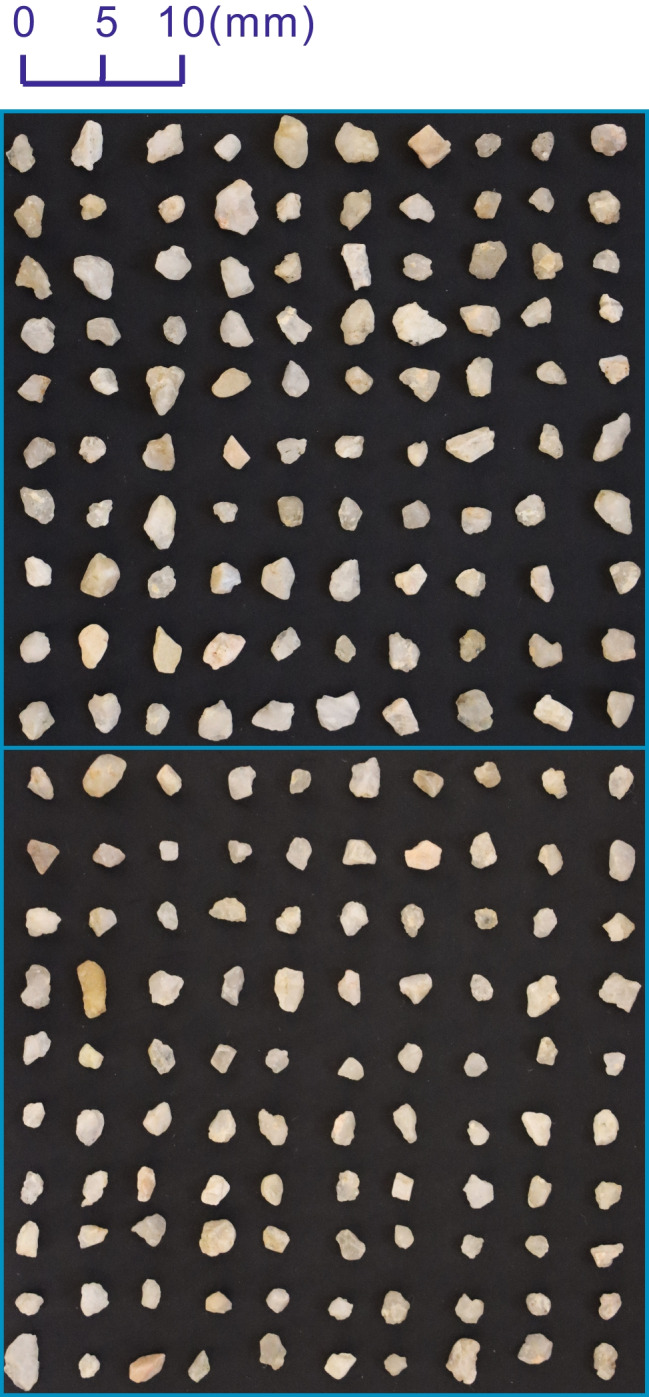
Sand sample of RSII

**Fig. 6:**
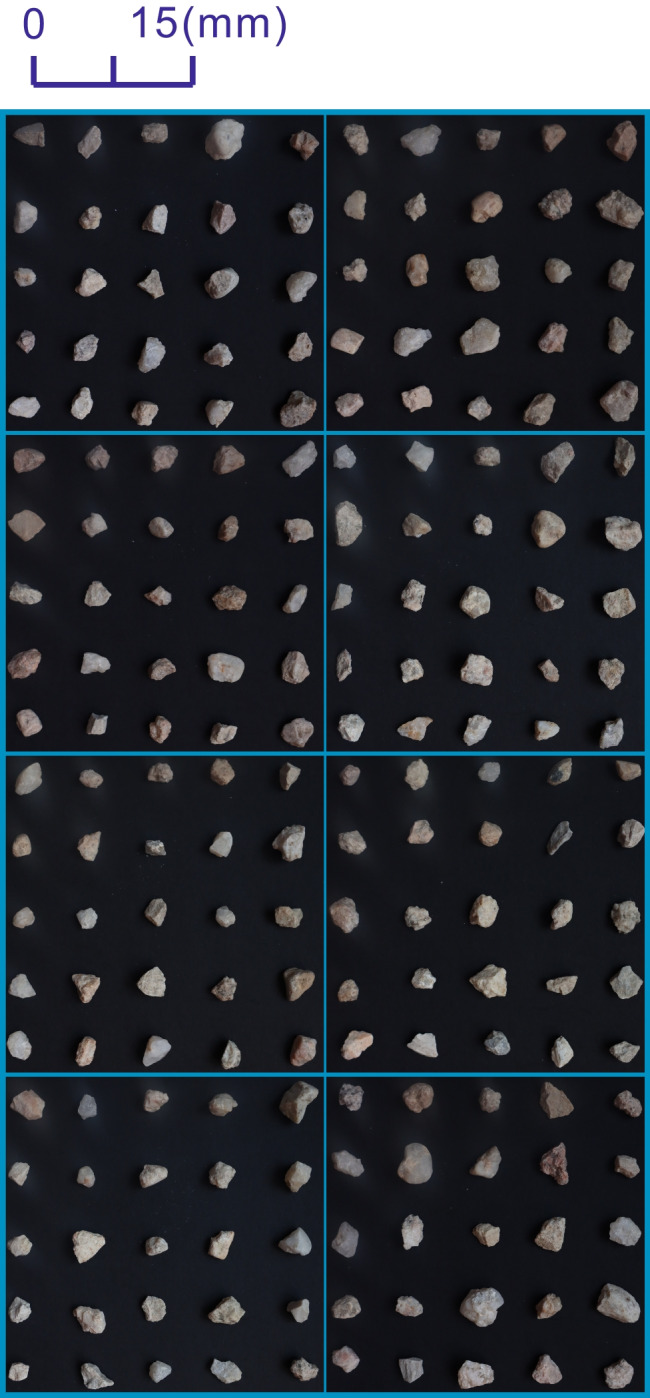
Sand sample of MS

The sand particles collected for this study encompass natural sands from the Hutuo River, the main water resource of Shijiazhuang County, Hebei Province, and manufactured crushed sands. The river sands are divided into 2 groups according to particle size: RS-I (particle size in 2–3 mm, see Fig. 4) and RSII (particle size in 3–4 mm, see Fig. 5). The particle size of the manufactured sands, marked as MS (see Fig. 6), varies from 3 to 10 mm. RSI, RSII, and MS consist of 200 particles, respectively.

## Results and discussion

### Analysis of shape descriptors

**Fig. 7:**
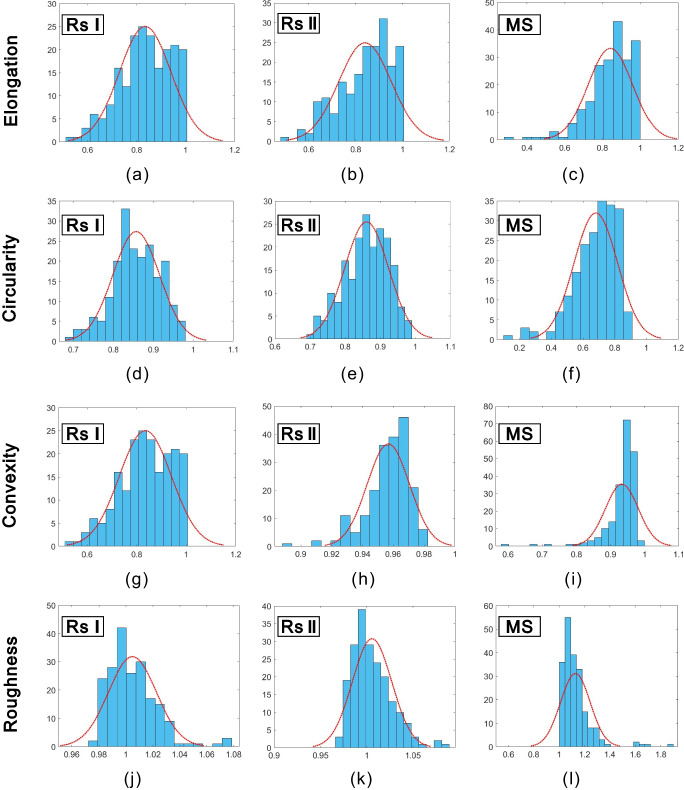
Frequency distribution histograms of elongation circularity convexity and roughness for RSI, RSII, and MS

**Fig. 8:**
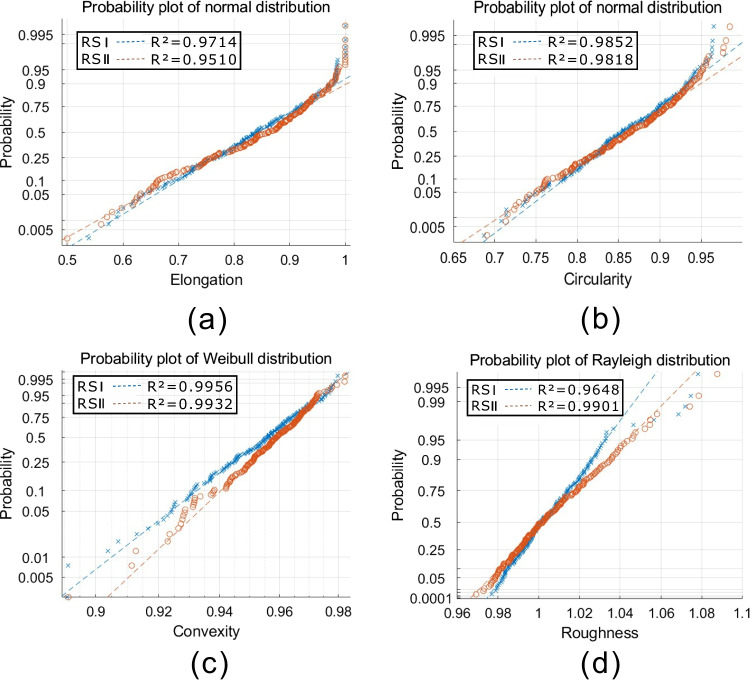
Probability plots of elongation circularity convexity and roughness for RSI and RSII

**Fig. 9:**
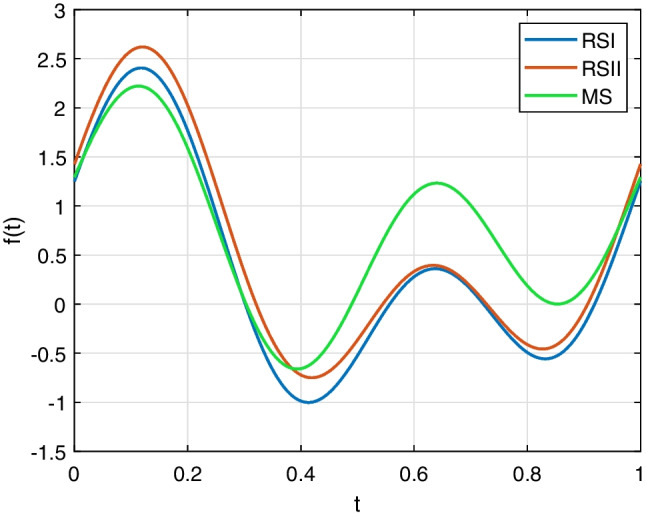
Andrews plot of elongation circularity convexity and roughness for RSI, RSII, and MS

The shape descriptors, elongation, circularity, convexity, and roughness, have been obtained using Eqs. (1) to (4), respectively, from 600 sand particles of RSI, RSII, and MS. Then, frequency distribution histograms for the four shape descriptors were plotted in Fig. 7. As can be seen from the figure, frequency distribution histograms of RSI and RSII are both skewed, while irregular distributions occurred for MS. Moreover, probability plots for each shape descriptor of RSI and RSII were plotted in Fig. 8. It is clearly showed that both elongation and circularity obey normal distribution for RSI and RSII, while the convexity obeys Weibull distribution and the roughness subjects to Rayleigh distribution. It can be seen from Figs. 7 and 8 that the elongation, circularity, convexity, and roughness were found out to display a statistically similar pattern for both RSI and RSII, while those for MS were found out to obey a statistically different pattern. The same statistical distribution models are also found in related literatures (Blott and Pye 2008; Yang et al. 2018), which mainly result from different formation processes. The Andrews plot of the four shape descriptors also showed that the Andrews curves of RSI and RSII blend into each other (Fig. 9). Thus, it is clear that the RSI, RSII, and MS can be divided into two types of sands: natural sand for RSI and RSII and manufactured sand for MS by elongation, circularity, convexity, and roughness.

### Analysis of Fourier descriptors

**Fig. 10:**
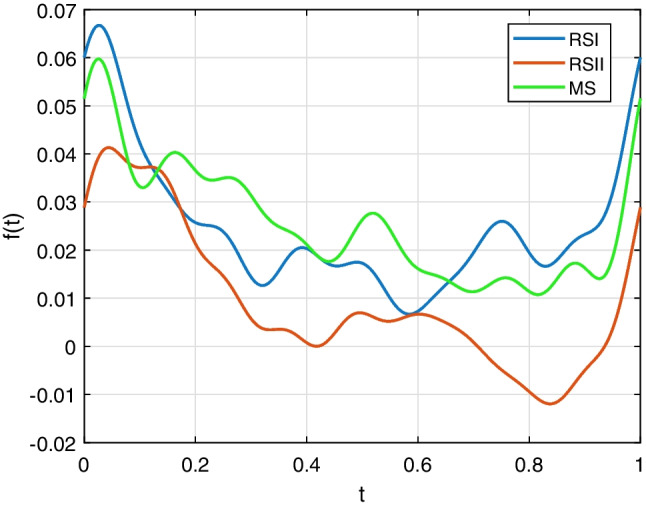
Andrews plot of Fourier descriptors for *D*_*1*_ to *D*_*18*_

The Fourier descriptors of RSI, RSII, and MS were calculated and then normalized based on the programs written by ourself. *D*_*0*_ was set to 0 because it only depends on the initial position. Then, the Andrews plot of the mode Fourier descriptors for *D*_*1*_ to *D*_*18*_ was plotted in Fig. 10. As can be seen, there are very few intersection points between the three Andrews curves of RSI, RSII, and MS. It leads to a conclusion that the Fourier descriptors can adequately discriminate the sand samples.

**Fig. 11:**
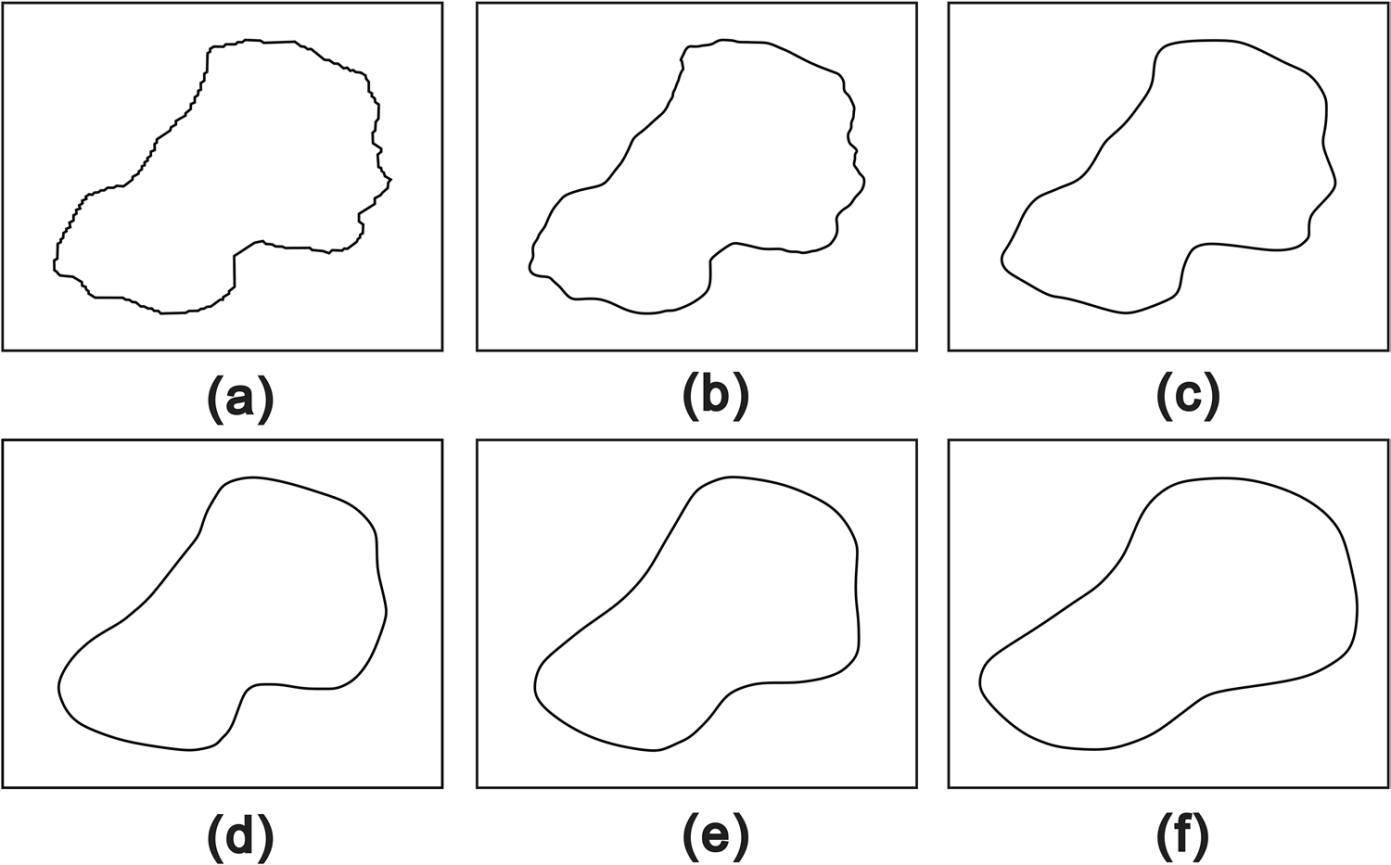
Particle boundary reconstructed using different number of Fourier descriptors. **a** Original boundary. **b**–**f** Boundaries reconstructed using 178, 36, 18, 12, and 10 Fourier descriptors, respectively

**Fig. 12:**
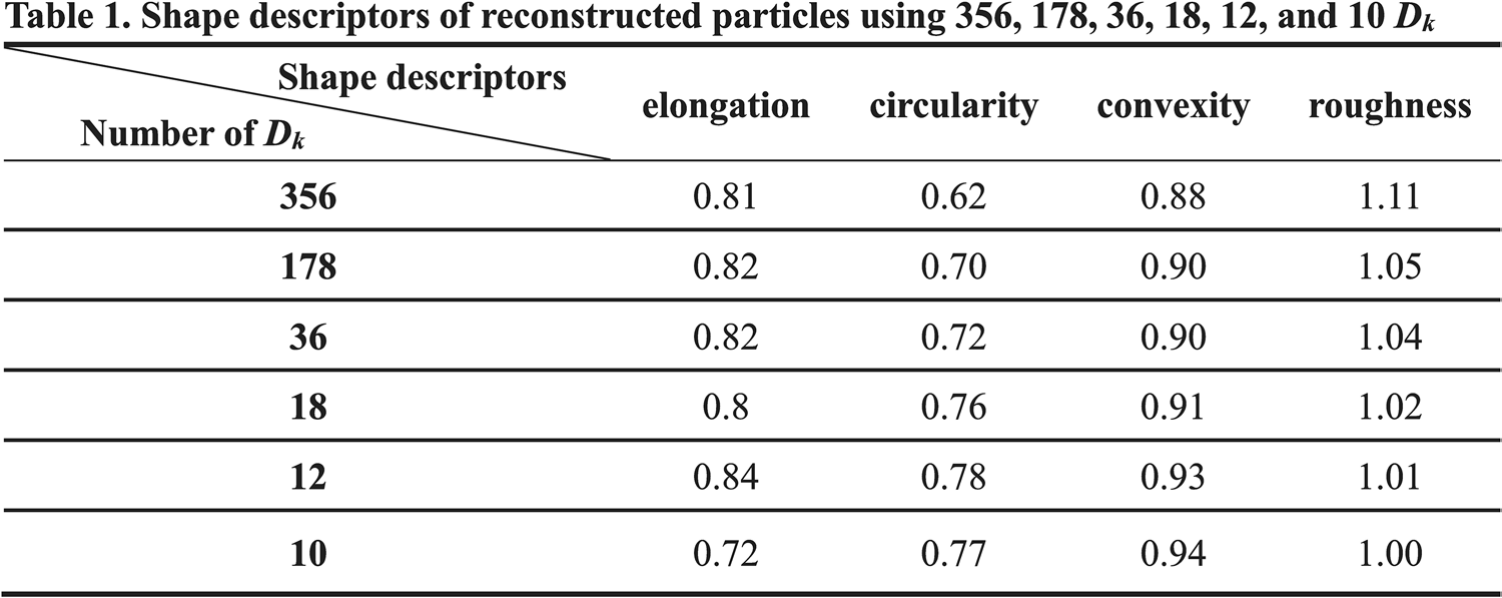
Shape descriptors of particles in Fig. 11

**Fig. 13:**
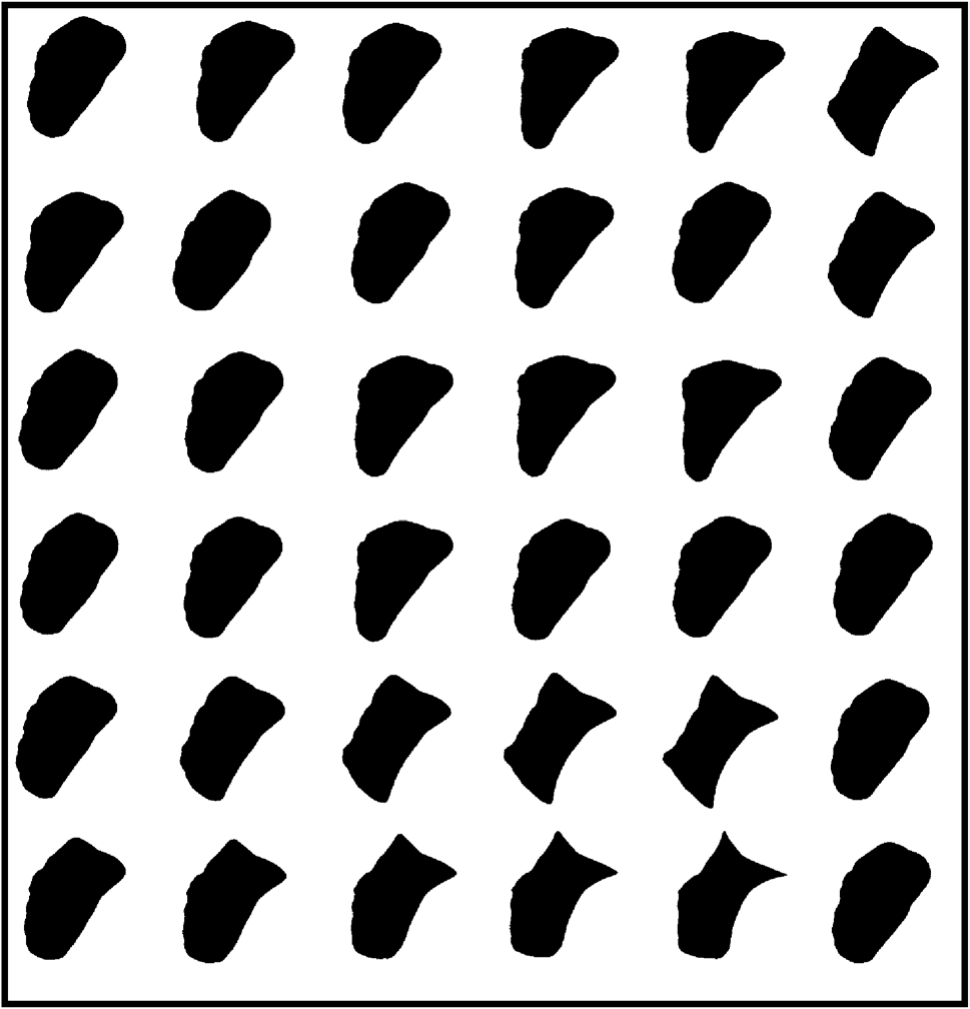
Particles reconstructed using Fourier descriptors

On the other hand, it has been known that Fourier descriptors are quite convenient for shape retrieval and reconstruction of the particles (Yang and Yu 2019; Mollon and Zhao 2013). The boundary of a sand particle from RSI, as shown in Fig. 11a, consists of 356 points. The corresponding 356 Fourier descriptors were obtained using Eq. (8). Figure 11b shows the boundary reconstructed using one-half of the 356 Fourier descriptors by the IFFT in Eq. (8). It is clear that there is no significant difference between this boundary and the original. Figures 11c through f show the boundaries reconstructed with the number of Fourier descriptors being 36, 18, 12, and 10, respectively. Figure 12 shows the shape descriptors of particles in Fig. 11. The more Fourier descriptors used in reconstruction, the generated particles are more similar to the original ones, which is also proved by the shape descriptors quantitatively. An algorithm to reconstruct particle boundary was proposed using all of the Fourier descriptors of a sand particle (Mollon and Zhao 2013). However, it turns out to be very time-consuming when simulating realistic geotechnical materials, which generally consist of thousands of particles. This study indicates that there is no need to use all of the Fourier descriptors when simulating realistic materials. However, it is not suggested to adopt less than 18 Fourier descriptors. Figure 13 shows 36 particles reconstructed using one quarter of the Fourier descriptors.

**Fig. 14:**
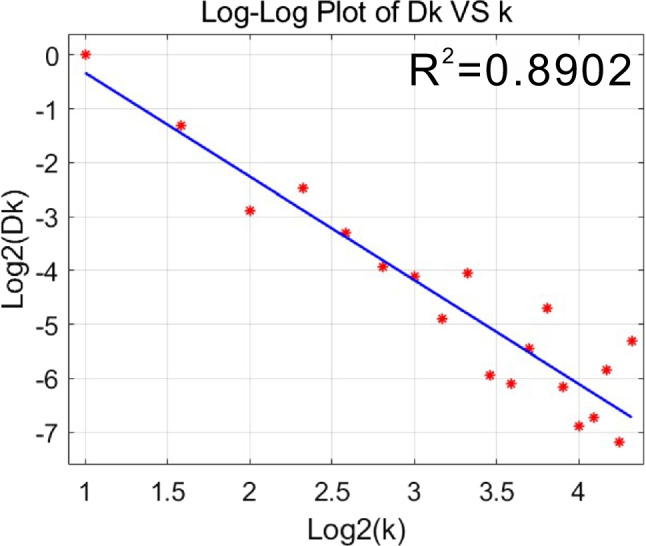
Log–Log plot of *D*_*k*_ versus *k*

Furthermore, Meloy proposed that there is a functional relationship between Fourier descriptors *D*_*k*_ and the frequency of the *k*th component (Meloy 1977). The sand particle in Fig. 11 was used to calculate the Fourier descriptors, and then the logarithms of the obtained *D*_*k*_ and *k* were taken to the base 2. And Fig. 14 shows a log–log plot of *D*_*k*_ and *k*; it is clear that there is a linear relationship between log2(*D*_*k*_) and log2(*k*).

### Shape descriptors versus Fourier descriptors

**Fig. 15:**
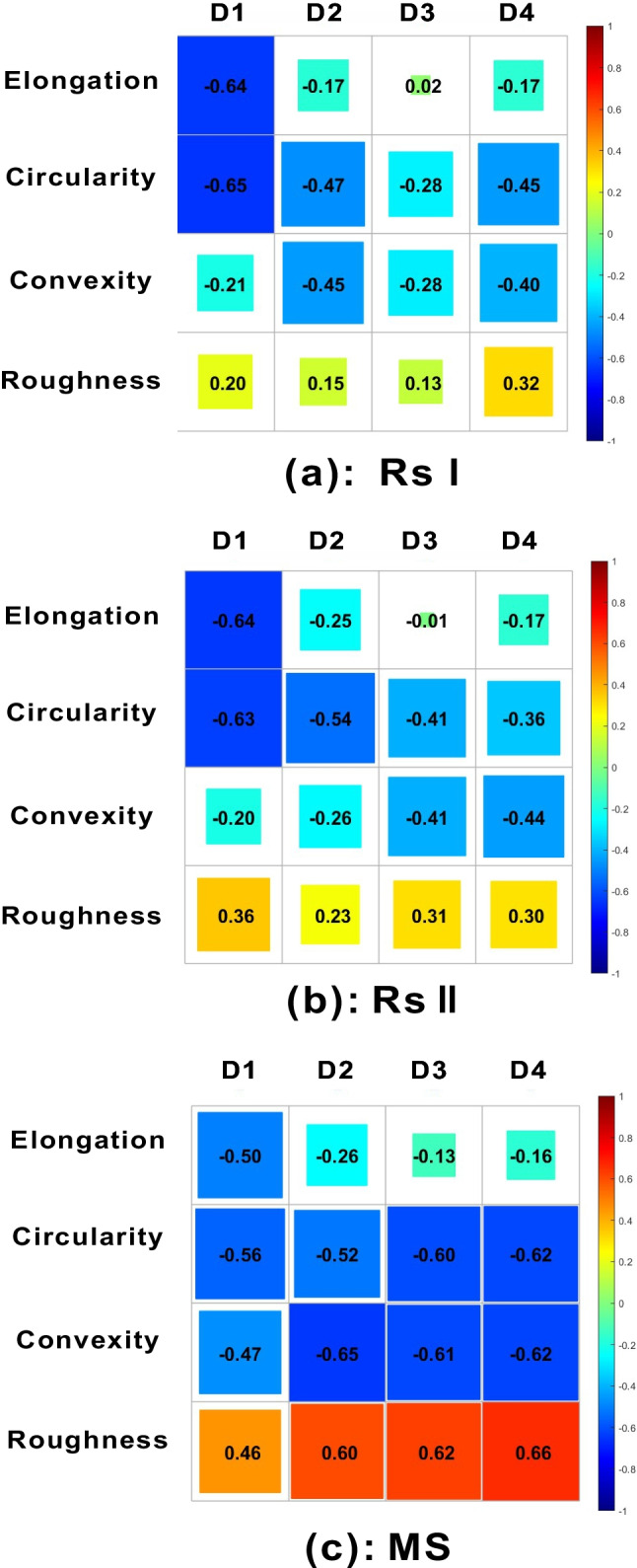
Correlation coefficients matrix plots between shape descriptors and Fourier descriptors

In order to assess the relationship between shape descriptors and Fourier descriptors, Pearson’s correlation coefficients were calculated for each kind of shape descriptors and Fourier descriptors of *D*_*1*_ through *D*_*4*_. The correlation coefficient matrix was plotted in Fig. 15, where Pearson correlation coefficients are presented as different color gradients, from red (absolute positive correlation: correlation coefficient 1) to blue (absolute negative correlation: correlation coefficient − 1). Note that elongation and circularity have a moderate negative correlation with *D*_*1*_ of RSI and RSII, coefficient *r* ranging from − 0.63 to − 0.65. As for MS, circularity and convexity show a moderate negative correlation with *D*_*2*_, *D*_*3*_, and *D*_*4*_, coefficient r ranging from − 0.62 to − 0.65, and roughness shows a moderate positive correlation with *D*_*2*_, *D*_*3*_, and *D*_*4*_, coefficient *r* ranging from 0.60 to 0.66.

**Fig. 16:**
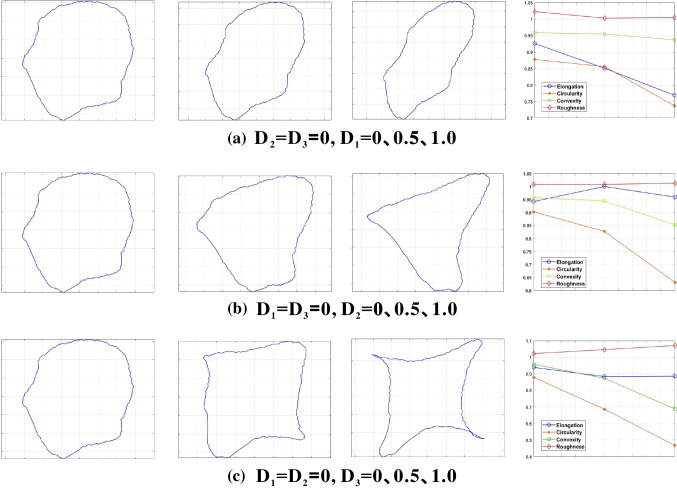
Relationship between shape descriptors and Fourier descriptors

**Fig. 17:**
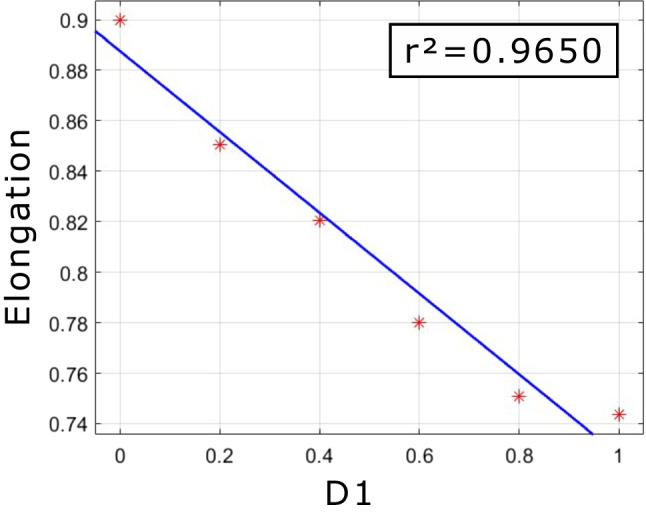
Relationship between elongation and *D*_*1*_

**Fig. 18:**
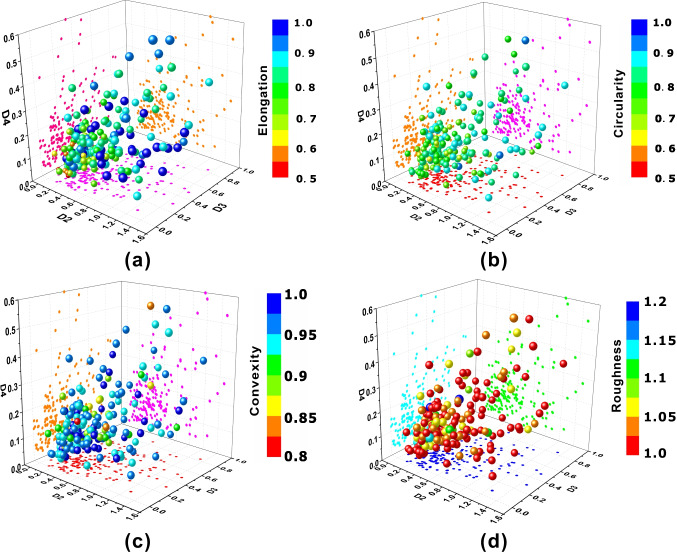
3D-scatter-diagram of *D*_*2*_, *D*_*3*_, *D*_*4*_, and shape descriptors

To further find out the relationship between shape descriptors and Fourier descriptors, a set of Fourier-descriptors-controlled experiments was conducted. A grain of sand was taken to calculate its Fourier descriptors. First of all, *D*_*1*_, *D*_*2*_, and *D*_*3*_ were set to 0. Then, two of the three Fourier descriptors were kept constant, and the remaining Fourier descriptor increased from 0 to 0.5, then to 1.0. Hence, 9 grains of sand were obtained, and then their shape descriptors, elongation, circularity, convexity, and roughness, were calculated, which can be seen in Fig. 16. It can be seen that the elongation decreased linearly as *D*_*1*_ increased, while the circularity and the convexity decreased in a nonlinearly manner as *D*_*1*_*, D*_*2*_, and *D*_*3*_ increased. But there is no significant change in roughness. Figure 17 furtherly proved that there exhibits a moderate negative linear correlation between *D*_*1*_ and elongation. Figure 18 indicates the statistic distribution of Fourier descriptors for *D*_*2*_, *D*_*3*_, and *D*_*4*_ and their relationship with elongation, circularity, convexity, and roughness. It can be seen that *D*_*2*_ varies from 0 to 0.6, *D*_*3*_ varies from 0 to 0.6, and *D*_*4*_ varies from 0 to 0.3. The elongation, circularity, convexity, and roughness were comparative evenly distributed.

## Conclusion

Based on the programs written by ourselves, this paper studies shape descriptors, Fourier descriptors, and their relationship of both natural and manufactured sand particles, as well as the method to simulate realistic particles using Fourier descriptors. The main conclusions could be drawn:

- Elongation and circularity obey normal distribution for natural sands, while the convexity obeys Weibull distribution and roughness subjects to Rayleigh distribution. And irregular distribution occurs to manufactured sands.
- Fourier descriptors provide comprehensive quantitative information about sand particles. And an algorithm is proposed to simulate sand particles using Fourier descriptors.
- Elongation shows a moderate negative correlation with Fourier descriptors of *D*_*1*_ for both natural and manufactured sands, and roughness shows a moderate positive correlation with Fourier descriptors of *D*_*2*_*, D*_*3*_, and* D*_*4*_ for manufactured sands.

Despite the above findings, this paper raises several questions that need to be explored:

- The Fourier descriptors were used to divide sand samples adopted in this paper very well. But its universality needs further verification.
- Although Fourier descriptors provide comprehensive information about sand particles, whether they contain more physical meanings needs further study to verify.
- A method is proposed to reconstruct sand particles using the Fourier descriptors. However, how to simulate the complex structure of geotechnic materials deserves more study.

## Data Availability

The data and materials generated and/or analyzed during the current study are available from the corresponding author on reasonable request.

## References

[CR1] Altuhafi FN, Coop MR (2011). Changes to particle characteristics associated with the compression of sands[J]. Géotechnique.

[CR2] Blott SJ, Pye K (2008). Particle shape: a review and new methods of characterization and classification[J]. Sedimentology.

[CR3] Bu S, Li D, Chen S (2022). Numerical simulation of landslide-generated waves using a SPH-DEM coupling model[J]. Ocean Eng.

[CR4] Das N (2007) Modeling three-dimensional shape of sand grains using discrete element method. USF Tampa Graduate Theses and Dissertations. https://digitalcommons.usf.edu/etd/689

[CR5] Gao W, Yang H, Wang L (2021). Numerical simulations of the soil–rock mixture mechanical properties considering the influence of rock block proportions by pfc2d[J]. Materials.

[CR6] Gonzalez R C (2009) Digital image processing[M]. Pearson education india, New York, pp 835–838

[CR7] Guo P, Su X (2007). Shear strength, interparticle locking, and dilatancy of granular materials[J]. Can Geotech J.

[CR8] Janoo V C (1998) Quantification of shape, angularity, and surface texture of base course materials[J]. U.S. Army Cold Regions Research and Engineering Laboratory: Special Report, pp 98–1

[CR9] Mair K, Frye KM, Marone C (2002). Influence of grain characteristics on the friction of granular shear zones[J]. J Geophys Res: Solid Earth.

[CR10] Meloy TP (1977). A hypothesis for morphological characterization of particle shape and physiochemical properties[J]. Powder TechnoL.

[CR11] Mollon G, Zhao J (2012). Fourier–Voronoi-based generation of realistic samples for discrete modelling of granular materials[J]. Granular Matter.

[CR12] Mollon G, Zhao J (2013). Generating realistic 3D sand particles using Fourier descriptors[J]. Granular Matter.

[CR13] Sokic E, Konjicija S (2016). Phase preserving Fourier descriptor for shape-based image retrieval[J]. Sig Process: Image Commun.

[CR14] Suh HS, Kim KY, Lee J (2017). Quantification of bulk form and angularity of particle with correlation of shear strength and packing density in sands[J]. Eng Geol.

[CR15] Suhr B, Six K (2020). Simple particle shapes for DEM simulations of railway ballast: influence of shape descriptors on packing behavior[J]. Granular Matter.

[CR16] Suhr B, Skipper WA, Lewis R (2020). Shape analysis of railway ballast stones: curvature-based calculation of particle angularity[J]. Sci Rep.

[CR17] Tsomokos A, Georgiannou VN (2010). Effect of grain shape and angularity on the undrained response of fine sands[J]. Can Geotech J.

[CR18] Wang J, Qian W, Chen G (2021). Combining quantitative analysis with an elliptic Fourier descriptor: a study of pottery from the Gansu-Zhanqi site based on 3D scanning and computer technology[J]. J Archaeol Sci Rep.

[CR19] Xu WJ, Dong XY (2021). Simulation and verification of landslide tsunamis using a 3D SPH-DEM coupling method[J]. Comput Geotech.

[CR20] Xu WJ, Xu Q, Liu GY (2021). A novel parameter inversion method for an improved DEM simulation of a river damming process by a large-scale landslide[J]. Eng Geol.

[CR21] Yang J, Luo XD (2015). Exploring the relationship between critical state and particle shape for granular materials[J]. J Mech Phys Solids.

[CR22] Yang C, Yu Q (2019). Multiscale Fourier descriptor based on triangular features for shape retrieval[J]. Sign Process: Image Commun.

[CR23] Yang J, Yu W, Fang H (2018). Detection of size of manufactured sand particles based on digital image processing[J]. PLoS ONE.

[CR24] Yang Y, Wei Z, Fourie A (2019). Particle shape analysis of tailings using digital image processing[J]. Environ Sci Pollut Res.

